# The prognostic value of DNA damage level in peripheral blood lymphocytes of chemotherapy-naïve patients with germ cell cancer

**DOI:** 10.18632/oncotarget.12515

**Published:** 2016-10-07

**Authors:** Zuzana Sestakova, Katarina Kalavska, Lenka Hurbanova, Dana Jurkovicova, Jan Gursky, Michal Chovanec, Daniela Svetlovska, Vera Miskovska, Jana Obertova, Patrik Palacka, Katarina Rejlekova, Zuzana Sycova-Mila, Silvia Cingelova, Stanislav Spanik, Jozef Mardiak, Miroslav Chovanec, Michal Mego

**Affiliations:** ^1^ Department of Genetics Cancer Research Institute, Biomedical Research Center, Slovak Academy of Sciences, Bratislava, Slovakia; ^2^ Translational Research Unit, Faculty of Medicine, Comenius University, National Cancer Institute, Bratislava, Slovakia; ^3^ 2nd Department of Oncology, Faculty of Medicine, Comenius University and National Cancer Institute, Bratislava, Slovakia; ^4^ Department of Oncology, National Cancer Institute, Bratislava, Slovakia; ^5^ 1st Department of Oncology, Faculty of Medicine, Comenius University and St. Elisabeth Cancer Institute, Bratislava, Slovakia; ^6^ Department of Oncology, St. Elizabeth Cancer Institute, Bratislava, Slovakia

**Keywords:** DNA damage, DNA repair, cisplatin, germ cell tumors, prognostic marker

## Abstract

Germ cell tumors (GCTs) are extraordinarily sensitive to cisplatin (CDDP)-based chemotherapy. DNA damage represents one of the most important factors contributing to toxic effects of CDDP-based chemotherapy. This study was aimed to evaluate the prognostic value of DNA damage level in peripheral blood lymphocytes (PBLs) from chemo-naïve GCT patients. PBLs isolated from 59 chemotherapy-naïve GCT patients were included into this prospective study. DNA damage levels in PBLs were evaluated by the Comet assay and scored as percentage tail DNA by the Metafer-MetaCyte analyzing software. The mean ± SEM (standard error of the mean) of endogenous DNA damage level was 5.25 ± 0.64. Patients with DNA damage levels lower than mean had significantly better progression free survival (hazard ratio [HR] = 0.19, 95% CI (0.04 – 0.96), *P* = 0.01) and overall survival (HR = 0.00, 95% CI (0.00 – 0.0), *P* < 0.001) compared to patients with DNA damage levels higher than mean. Moreover, there was significant correlation between the DNA damage level and presence of mediastinal lymph nodes metastases, IGCCCG (International Germ Cell Cancer Collaborative Group) risk group, and serum tumor markers level. These data suggest that DNA damage levels in PBLs of GCT patients may serve as an important prognostic marker early identifying patients with poor outcome.

## INTRODUCTION

Germ cell tumors (GCTs) are the most common cancer disease seen in young men between 20-40 years of age [1]. GCTs also represent highly curable malignancy. Nearly 80% of GCT patients with metastatic disease may be cured by cisplatin (CDDP)-based chemotherapy [2]. However, approximately 20% of patients do not reach complete remission and suffer from relapse of the disease. Patients who fail to be cured after salvage chemotherapy have an extremely poor prognosis [3]. Therefore, understanding the molecular mechanism(s) implicated in germ cell cancer therapy failure may represent the key tool for more effective treatment [4].

The mechanisms underlying CDDP sensitivity/resistance are determined by a variety of factors including a major for the DNA damage response and repair. CDDP is classified as a DNA-damaging alkylating agent. It forms monoadducts with purines (preferred site is the N7 position of guanines), intrastrand cross-links (IaCLs) with 1,2-IaCL and 1,3-IaCL representing 90% of CDDP lesion burden, interstrand cross-links (ICLs) occurring principally at d(GpC):d(GpC) sites, accounting for only 5% of all adducts, and protein-DNA cross-links. Although all these individual lesions contribute to the toxicity of CDDP, there is compelling evidence that ICLs are the critical cytotoxic CDDP-induced lesions [5].

Due to the extraordinary sensitivity of GCTs to CDDP, it has been assumed that limited DNA repair may be responsible for unique curability of this type of cancer [6, 7]. However, it is not clear yet whether GCTs become resistant to this drug *via* increasing DNA repair capacity, since the data reported so far are rather inconclusive [7, 8, 9]. Structurally, CDDP-induced ICLs can cause extrusion of cytosines, complementary to the adduced guanines, from DNA helix and these structures resemble substrates that are ordinarily recognized by the mismatch repair (MMR) factors [10]. Hence, it is not surprising that several studies have been aimed to determine a role of MMR in GCTs, but present data are also rather controversial in terms of presence of microsatellite instability regions arising as a consequence of MMR defects in this type of malignancy [11, 12, 13, 14, 15, 16]. Moreover, CDDP-induced DNA lesions are detected and removed by action of nucleotide excision repair (NER). Therefore, an association between GCTs and the two key NER factors, XPA (DNA lesion recognition factor) and ERCC1/XPF (structure-specific endonuclease complex), has been extensively examined: lower levels of both factors have indeed been linked with CDDP sensitivity in several cell lines derived from GCTs [7, 17, 18]. However, there is an evidence that besides its role in NER, ERCC1/XPF participates in other DNA repair processes such as ICL repair and certain homologous recombination (HR) events where it facilitates DNA double-strand break (DSB) repair [19, 20], and thus this repair factor may obviously affect CDDP response in a pleiotropic manner through different DNA repair pathways. Within HR, two genes, *XRCC2* and *RAD51C*, have been reported to be associated with GCTs [21, 22]. Moreover, it has been shown that reduced HR repair corresponds with either incapability of, or reduced ability to, repair CDDP-induced DNA damage in several GCT cell lines [23]. Data on cellular level also revealed a role of Fanconi anemia (FA) pathway and translesion DNA synthesis (TLS) in CDDP response, although these have not been examined in GCTs yet.

It has been proposed that targeted inhibition of relevant DNA repair factors could sensitize tumors to therapy. Therefore, information on DNA repair capacity may represent a potential, and even essential, biomarker for cancer treatment in the future. In line with this assumption, data evaluating DNA repair capacity in peripheral blood lymphocytes (PBLs) isolated from cancer patients suggested that functional assays for DNA repair protein/enzyme activity, like the Comet assay, provide much more useful and clinically relevant information than measuring expression of the DNA repair genes *per se* [24, 25]. The Comet assay is relatively simple, sensitive, rapid and inexpensive method that has already been employed in several DNA damage and repair clinical studies, where PBLs was used as a tumor surrogate [25, 26]. The aim of our study was to investigate the level of endogenous DNA damage (potentially arising as a consequence of aberrant DNA repair capacity) in PBLs from chemo-naïve GCT patients using the Comet assay in order to address the question whether it could possibly be used as a prognostic factor in this malignity.

## RESULTS

### Patients characteristics

Analyzed cohort consisted of 59 chemotherapy-naïve GCT patients before starting CDDP-based chemotherapy treated in the National Cancer Institute of Slovakia and St. Elisabeth Cancer Institute in Bratislava, Slovakia. Basic and clinical patients' characteristics are summarized in Table 1 and Supplementary Table S1, respectively. The median age of patients enrolled into this study was 32 years (ranging from 18 to 60 years). The majority of patients had non-seminomatous primary testicular tumor and a good prognosis according to IGCCCG (International Germ Cell Cancer Collaborative Group). Tumor specimen included 17 pure seminomas, 11 non-seminomas (5 embryonal carcinomas, 2 yolk sac tumors, 3 choriocarcinomas and 1 teratoma) and 29 mixed GCTs (Supplementary Table S2).

**Table 1:** Patients' characteristics (n = 59)

	Chemotherapy-naïve GCTs	
	N = 59	%
**Age (years)**		
Median (range)	32 (18-60)	
**Primary tumor**		
Gonadal	53	89.8
Extragonadal - retroperitoneal	4	6.8
Extragonadal - mediastinal	2	3.4
**Histology**		
Seminoma	18**^a^**	30.5
Non-seminoma	39**^a^**	66.1
**Stage of GCTs**		
Stage I.A-I.B	11	18.6
Stage I.S	2	3.4
Stage II.A-III.A	32	54.2
Stage III.B	8	13.6
Stage III.C	6	10.2
**Sites of metastases**		
Retroperitoneum	41	69.5
Mediastinum	5	8.5
Lung	9	15.3
Liver	3	5.1
Brain	1	1.7
Other	2	3.4
Visceral non-pulmonary mts	5	8.5
**IGCCCG risk group**		
Good prognosis	45	76.3
Intermediate prognosis	8	13.6
Poor prognosis	6	10.2
**Mean (range) of pretreatments markers**		
AFP mIU/ml	234.1 (0.0-5810.0)**^b^**	
HCG IU/ml	107104.0 (0.0-1840510.0)**^b^**	
LDH (mkat/l)	8.6 (1.4-57.3)**^c^**	

a histology data are not available for 2 patients

b data available for 43 patients

c data available for 42 patients

Fifty (84.7%) of tested patients were treated with BEP (bleomycin, etoposide, CDDP) regimen and nine patients (15.3%) received EP (etoposide, CDDP) chemotherapy. All patients received G-CSF (granulocyte-colony stimulating factor) support (filgrastim or pegfilgrastim) after chemotherapy. In addition, four (6.8%) patients from the studied cohort underwent radiation therapy.

### Association between DNA damage levels and patients/tumor characteristics

The mean ± SEM (standard error of the mean) of endogenous DNA damage level in PBLs from chemo-naïve GCT patients was 5.25 ± 0.64. Statistical analysis showed no significant association between the mean DNA damage level in lymphocytes and patients/tumor characteristics, including tumor primary, retroperitoneal lymph nodes metastases, lung metastases, and/or non-pulmonary visceral metastases. However, a significant correlation between the DNA damage level in PBLs and IGCCCG risk group (*P* = 0.02), as well as mediastinal lymph nodes metastases (*P* < 0.001) or S-stage of disease (*P* < 0.001) was found (Table 2).

**Table 2:** Association between the Comet assay and patients/tumor characteristics in chemotherapy-naïve GCT patients (n =59)

Variable	N	The Comet assay							
		Mean	SEM	*P*-value	<mean = 5.25		>mean = 5.25		*P*-value
					N	%	N	%	
**All patients**	59	5.25	0.60	NA	44	100.0	15	100.0	NA
Tumor primary*									
Seminoma	18	4.75	1.12	0.41	13	29.5	5	33.3	0.75
Non-seminoma	39	5.36	0.75		30	68.2	9	60.0	
**IGCCCG risk group**									
Good and intermediate prognosis	53	5.11	0.64	0.02	41	93.2	12	80.0	0.59
Poor prognosis	6	6.49	1.89		3	6.8	3	20.0	
**Retroperitoneal lymph nodes metastases**									
Absent	18	4.38	1.09	0.25	16	36.4	2	13.3	0.12
Present	41	5.63	0.72		28	63.6	13	86.7	
**Mediastinal lymph nodes metastases**									
Absent	54	5.17	0.63	0.06	42	95.5	12	80.0	<0.001
Present	5	6.14	2.07		2	4.5	3	20.0	
**Lung metastases**									
Absent	50	5.25	0.66	0.28	39	88.6	11	73.3	0.21
Present	9	5.26	1.55		5	11.4	4	26.7	
**Non-pulmonary visceral metastases**									
Absent	54	5.19	0.63	0.09	41	93.2	13	86.7	0.59
Present	5	5.90	2.08		3	6.8	2	13.3	
**S – stage**									
0-2	55	5.08	0.62	0.01	43	97.7	12	80.0	<0.001
3	4	7.51	2.30		1	2.3	3	20.0	

* histology data are not available for 2 patients, SEM – standard error of mean, NA – not applicable

### Prognostic value of DNA damage level in lymphocytes

**Figure 1:**
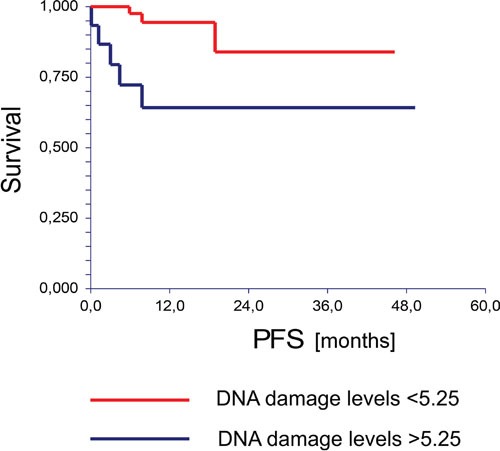
Kaplan-Meier estimates of probabilities of progression free survival according to the Comet assay in chemotherapy-naïve GCT patients (n = 59), HR = 0.19, 95% CI (0.04-0.96), P = 0.0101

**Figure 2:**
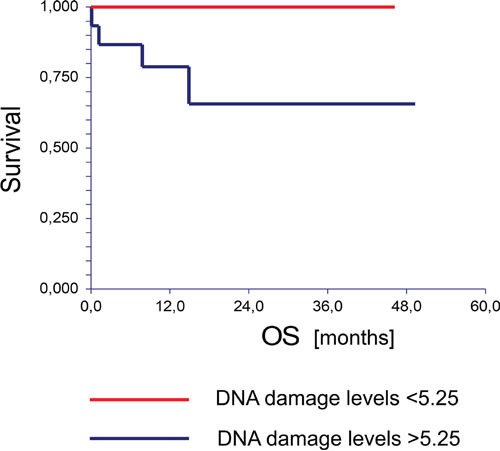
Kaplan-Meier estimates of probabilities of overall survival according to the Comet assay in chemotherapy-naïve GCT patients (n = 59), HR = 0.00, 95% CI (0.00-0.00), P = 0.0006

The median follow-up was 11.9 months (range 0.1 – 49.3 months). By the end of this period, eight (13.6%) patients experienced disease progression and four (6.8%) patients died. Univariate analysis of tested cohort showed that patients with DNA damage levels in lymphocytes lower than the mean value had significantly better PFS compared to patients with DNA damage levels higher than the mean value (hazard ratio [HR] = 0.19, 95% CI (0.04 – 0.96), *P* = 0.01) (Figure 1). Moreover, the OS of chemo-naïve GCT patients significantly correlated with DNA damage levels in lymphocytes of the studied patients (HR = 0.00, 95% CI (0.00 – 0.0), *P* = < 0.001) (Figure 2). Using the median value 4.24 to dichotomize the analyzed patients, we observed the similar association of DNA damage levels and PFS (HR = 0.14, 95% CI (0.03 – 0.55), *P* = 0.03) or OS (HR = 0.00, 95% CI (0.00 – 0.0), *P* = 0.05).

In multivariate analysis, we revealed that DNA damage levels in patients' lymphocytes were significantly associated with PFS and OS independently of IGCCCG risk group (Table 3).

**Table 3:** Multivariate analysis

Variable	PFS		OS	
	HR (95% CI)	*P*-value	HR (95% CI)	*P*-value
**Comet assay (DNA damage level) high *vs*. low**	10.32 (5.40-19.73)	0.0049	> 100 (NA)	0.0022
**IGCCCG risk group poor *vs*. good/intermediate prognosis**	35.60 (14.00 – 90.57)	< 0.0001	18.15 (7.03-46.90)	0.0067

## DISCUSSION

In this prospective study, we sought to define the association between DNA damage levels in PBLs of chemotherapy-naïve GCT patients and the patients' outcome. Patients with high DNA damage levels in PBLs before chemotherapy had significantly worse PFS and OS compared to patients with low DNA damage levels, as measured by the Comet assay. Moreover, no patients with low DNA damage levels died during the follow-up period suggesting that this assay could indeed identify GCT patients with extremely good prognosis. We also found significant correlation between the DNA damage levels and some negative prognostic features like IGCCCG high risk group, the presence of mediastinal lymph nodes metastases, and/or S-stage of disease; however, in multivariate analysis endogenous DNA damage levels were prognostic factor for PFS and OS independently of the IGCCCG prognostic group.

Introduction of CDDP-based chemotherapy dramatically improved the prognosis of patients with metastatic testicular cancer [27, 28] and DNA damage represents one of the most important factors contributing to toxic effects of this chemotherapy [29, 30, 31]. Hence, DNA repair capacity as well as DNA damage levels in tumor cells and/or PBLs may serve as potential predictive biomarkers for better stratification of GCT patients. Moreover, they may represent new specific targets for therapy of this disease.

Although our detailed knowledge on how DNA repair capacity corresponds with high curability of GCTs by CDDP is still largely unknown, valuable clues could be provided by reports on other cancer types. In general, aberrant DNA repair activity, due to defect in NER, base excision repair (BER), MMR, HR and TLS, has often and clearly been linked to CDDP-based therapy outcome in several malignancies [32, 33]. In the case of GCTs, non-seminomas display higher expression levels of the ERCC1 repair factor compared to seminomas and normal testicular tissue [34]. Elevated levels of ERCC1 are also observed in GCT cells resistant to CDDP [35]. On the other hand, XPA levels are lower in sensitive GCTs [17], indicating that expression levels of these two key NER factors may potentially be used as prognostic markers and potential biomarkers of CDDP response. Furthermore, they could serve as the basis for developing of clinically relevant strategies and therapeutic targets in treatment of GCTs resistant to CDDP [23].

HR factors including PARP (Poly(ADP-ribose)polymerase) can also modulate CDDP-related DNA repair capacity in GCTs [30]. It has been shown that PARP protein is significantly overexpressed in GCTs compared to normal testicular tissue [36] and promising target for therapeutic strategies based on a principle of synthetic lethality, where PARP inhibitors efficiently and selectively kill cells deficient in HR [37]. In context of GCTs, a reduced proficiency of HR has been reported as the basis of sensitivity of embryonal carcinoma cells to CDDP and monotherapy using PARP inhibitor. Hence, it has been proposed that PARP inhibitors might be used to implement GCT therapy, especially in patients resistant to standard therapies [30, 34]. That HR is strongly implicated in defining the response of GCTs to CDDP is well-documented by the fact that XRCC2, a protein promoting CDDP resistance *via* HR and FA pathways [38, 39, 40], has been found mutated in two GCT patients [22]. Interestingly, these mutations occurred in refractory patients, indicating that they confer resistance rather than sensitivity to chemotherapy [22]. In support, one of the two *XRCC2* mutations (R188H) indeed caused more tolerant phenotype to CDDP in DT40 cells [41].

Here, we show that DNA damage levels in chemo-naïve GCT patients are significantly associated with PFS and OS. These data suggest that the corresponding DNA repair capacity could be involved in prognosis of GCT patients. There are several possible explanations for this observation. One possibility is that testicular cancer cells have DNA repair capacity inherited from normal spermatogonial cells before their malignant transformation, and thus DNA damage levels in PBLs is a surrogate of DNA repair capacity observed in cancer cells. The Comet assay detects several DNA damage types, therefore we cannot specify which of the DNA repair pathways is responsible for this association. We hypothesize that increased DNA damage levels in patients with inferior outcome are associated with alterations in specific DNA repair pathways that subsequently lead to aberrant response to CDDP-based chemotherapy, however an exact mechanism remains to be elucidated. Another explanation is that increased DNA damage levels are responsible for higher mutation rate in germ cell cancer with subsequent higher chance of occurrence of resistant clone. Alternatively, DNA damage levels in PBLs could be unrelated to DNA repair capacity in cancer cells. Finally, patients with higher endogenous DNA damage levels could have an increased systemic toxicity and thus decreased dose intensity of chemotherapy; however, we have observed no differences in relative dose intensity of chemotherapy according to endogenous DNA damage levels in PBLs. Immune-checkpoint inhibitors are new class of anticancer agents with promising activity in various types of cancer [42]. Data suggest that increased mutational load in tumors is positively correlated with efficacy of these agents [43]. We suppose that GCT patients with higher endogenous DNA damage levels could be candidate for this therapy. It remains to be elucidated if high-dose chemotherapy with autologous stem cell support could overcome inferior outcome of GCT patients with higher endogenous DNA damage levels.

Previously, higher endogenous DNA damage levels were revealed in patients who develop non-seminoma compared to seminoma [44], although we have not confirmed this association. As suggested, this fact could explain more aggressive nature and younger age at diagnosis of non-seminoma compared with the relatively less aggressive, later onset seminoma. Importantly, DNA damage data presented here reflect mainly single-strand breaks (SSBs) and alkali-labile sites. SSBs can arise in DNA directly through action of reactive oxygen species [31, 45] and it has been known for a long time that cancer cells naturally display elevated levels of these lesions [31, 46]. However, they can also be generated indirectly as DNA repair intermediates, and thus aberrant DNA repair is a contributing factor. This possibility is currently being examined in our laboratory.

Beside certain strengths, this study has also some limitations including limited sample size and lack of validation cohort, thus the presented results are hypothesis-generating, and despite their biological and clinical rationale they should be confirmed in further prospective studies. Moreover, our results are applicable only to chemotherapy-naïve GCT patients and similar studies in relapsed/refractory patients are warranted. As the Comet assay measures a variety of DNA damage types, the identification of specific DNA repair pathways responsible for observed results as well as assessment of endogenous DNA damage in primary GCTs and its correlation to PBLs are warranted.

In conclusion, in this pilot study, for the first time to our knowledge, we show an association between DNA damage levels in PBLs of chemotherapy-naïve GCT patients and patients' outcome. Based on our data, we suggest that DNA damage levels in PBLs of GCT patients may potentially serve as an important prognostic marker associated with poor PFS and OS and after further validation could be used for better stratification of GCT patients for clinical trials. Hence, reliable methods for detecting DNA damage levels in PBLs in cancer patients may extend the diagnostic and prognostic tool set, and targeting DNA damage repair pathways may contribute to improving conventional therapy regimens.

## PATIENTS AND METHODS

### Study patients

The present study (Protocol IZLO1, Chair: M. Mego) involved 59 chemotherapy-naïve men with GCTs treated from May 2012 to June 2015 in the National Cancer Institute of Slovakia and/or St. Elisabeth Cancer Institute in Slovakia. Patients with concurrent malignancy other than non-melanoma skin cancer in the previous 5 years were excluded from the study. Adjuvant chemotherapy was administrated to 6 GCT patients (18.6%) enrolled into this study. Clinical stage of disease was determined according to the 2010 TNM (Tumor Node Metastasis) staging system [47]. Data regarding age, tumor histological subtype, clinical stage and type and number of metastatic lesions have been recorded in all patients. GCT patients were recruited and consented according to the Institutional Review Board approved protocol.

### Lymphocyte preparation

Peripheral blood samples were collected into lithium-heparin treated tubes (BD, Vacutainer Blood Collection Tubes) at baseline in the morning on day −1 or 0 of first cycle of chemotherapy. Lymphocytes were separated using Histopaque-1077 (Sigma-Aldrich, Germany), which mediates blood cells layering. After centrifugation, separated lymphocytes were washed twice with and resuspended in PBS at a cell density of 1×10^6^ cells/ml.

### Comet assay

The Comet assay was carried out as previously described [45]. Briefly, the lymphocyte suspension (approximately 1-2×10^4^ cells) was mixed with low melting point agarose and spread onto fully frosted microscopic slides covered with high melting point agarose. The prepared slides were kept at 4°C until the agarose solidified. After removal of coverslip, the cells were lysed in freshly prepared cold lysis solution (2.5 M NaCl, 10 mM Tris-HCl, 100 mM Na_2_EDTA, pH 10.0) with 1% Triton-X for 60 minutes at 4°C. Electrophoresis was applied in a horizontal gel electrophoresis tank filled with fresh electrophoresis buffer (0.2 mM Na_2_EDTA, 5 M NaOH) for 30 minutes at 4°C. Following electrophoresis, slides were neutralized in 1 M Tris-HCl for 15 minutes. Altogether, 100 randomly-selected cells *per* slide were analyzed through the Metafer-MetaCyte analyzing software (Metasystems, Altlussheim, Germany), and the level of DNA damage was expressed as % DNA in tail.

### Statistical analysis

The patients' characteristics were tabulated and summarized as mean (range) values for continuous variables and frequency (percentage) for categorical variables, respectively. Normality of distribution was tested by the Kolmogorov-Smirnoff test. If normally distributed, sample means were tested by Student *t*-test or analysis of variance (ANOVA) with Bonferroni's or Tamhane's corrections, depending on the homogeneity of variance. Nonparametric Mann-Whitney *U* or Kruskal-Wallis H-tests were used for non-normally distributed data, whereas Fisher's exact test was used when DNA damage was categorized as ‘low’ or ‘high’ according to the cut-off level of mean.

Median follow-up period was calculated as a median observation time of all patients, as well as of those still alive at the time of the last follow-up. Progression-free survival (PFS) was calculated from the date of the starting CDDP-based chemotherapy to date of progression or death, or date of the last adequate follow-up. Overall survival (OS) was calculated from date of starting systemic therapy to date of death or last follow-up. PFS and OS rates were estimated using the Kaplan-Meier product limit method and were compared with the log-rank test to determine significance.

To assess differences in survival (PFS, OS) based on the level of DNA damage in patients' lymphocytes and prognosis according to the IGCCCG criteria (1997) [48], a multivariate Cox proportional hazards model for PFS and OS was used. All presented *P*-values were two-sided. Values of *P* < 0.05 were considered as significant. Statistical analyses were performed using NCSS 10 software [49].

## SUPPLEMENTARY TABLES




