# Supplementary material for: The prognostic value of DNA damage level in peripheral blood lymphocytes of chemotherapy-naïve patients with germ cell cancer
Source: Oncotarget. 2016 Oct 7;7(46):75996–6005. doi: 10.18632/oncotarget.12515 (PMC5342793; doi:10.18632/oncotarget.12515)
Supplement: Supplementary file 1 [file oncotarget-07-75996-s001.pdf]

The prognostic value of DNA damage level in peripheral blood lymphocytes of chemotherapy-naïve patients with germ cell cancer

SUPPLEMENTARY TABLES

Supplementary Table S1: Clinical characteristics of study patients (n=59)

See Supplementary File 1

Supplementary Table S2: Distribution of histological subtypes among GCT patients (n=59)

Histological subtype*					Number of patients
SEM					17
	EC				5
	EC	YST			4
	EC	YST		TER	5
SEM	EC				3
		YST			2
			CHC		3
	EC			TER	2
SEM		YST			2
SEM	EC	YST			2
SEM	EC			TER	2
EC		YST	CHC	TER	2
				TER	1
		YST		TER	1
			CHC	TER	1
	EC		CHC	TER	1
SEM		YST		TER	1
SEM		YST	CHC		1
SEM	EC	YST		TER	1
SEM	EC	YST	CHC	TER	1

Abbreviations: EC, embryonal carcinoma; SEM, seminoma; YST, yolk sac tumour; CHC, choriocarcinoma; TER, teratoma
* Complete histology data are not available for 2 patients
