# Supplementary material for: The prognostic value of DNA damage level in peripheral blood lymphocytes of chemotherapy-naïve patients with germ cell cancer
Source: Oncotarget. 2016 Oct 7;7(46):75996–6005. doi: 10.18632/oncotarget.12515 (PMC5342793; doi:10.18632/oncotarget.12515)
Supplement: Supplementary file 2 [file oncotarget-07-75996-s002.doc]

**Supplementary table 1. Clinical characteristics of study patients (n=59)**

| **Patient no.** | **Age (years)** | **Histology** | **Primary tumor** | **Progression of disease** | **Death** | **Stage of GCTs** | **Radiotherapy** | **IGCCCG risk group** | **Retroperitoneal metastasis** | **Mediastinal metastasis** | **Lung metastasis** | **Liver metastasis** | **Brain metastasis** | **Visceral non-pulmonary metastasis** | **Other metastasis** | **DNA damage level in PBLs** |
| --- | --- | --- | --- | --- | --- | --- | --- | --- | --- | --- | --- | --- | --- | --- | --- | --- |
| **1** | 23 | non-seminoma | testis | + | + | III.C | - | 3 | + | - | + | + | - | + | + | 8.77 |
| **2** | 23 | not available | testis | + | + | III.C | - | 3 | + | - | + | + | - | + | - | 6.72 |
| **3** | 29 | not available | testis | + | + | III.B | - | 2 | + | - | - | - | - | + | - | 8.28 |
| **4** | 25 | non-seminoma | retroperitoneum | + | - | III.C | - | 3 | + | - | + | + | - | - | - | 5.10 |
| **5** | 39 | seminoma | testis | + | - | II.C | - | 1 | + | - | - | - | - | + | - | 4.81 |
| **6** | 28 | seminoma | mediatinum | + | - | III.C | + | 3 | - | - | - | - | + | - | - | 3.90 |
| **7** | 28 | non-seminoma | testis | - | - | I.B | - | 1 | - | - | - | - | - | - | - | 3.90 |
| **8** | 53 | non-seminoma | testis | - | - | I.B | - | 1 | - | - | - | - | - | - | - | 3.51 |
| **9** | 24 | non-seminoma | testis | - | - | I.A | - | 1 | - | - | - | - | - | - | - | 2.66 |
| **10** | 30 | non-seminoma | testis | - | - | I.A | - | 1 | - | - | - | - | - | - | - | 7.56 |
| **11** | 23 | non-seminoma | testis | - | - | I.B | - | 1 | - | - | - | - | - | - | - | 3.82 |
| **12** | 33 | non-seminoma | testis | - | - | I.B | - | 1 | - | - | - | - | - | - | - | 3.07 |
| **13** | 21 | non-seminoma | testis | - | - | I.B | - | 1 | - | - | - | - | - | - | - | 4.55 |
| **14** | 34 | non-seminoma | testis | - | - | II.B | - | 1 | + | - | - | - | - | - | - | 4.48 |
| **15** | 37 | non-seminoma | testis | - | - | II.A | - | 1 | + | - | - | - | - | - | - | 4.51 |
| **16** | 31 | non-seminoma | testis | - | - | II.B | - | 1 | + | - | - | - | - | - | - | 3.73 |
| **17** | 43 | seminoma | testis | - | - | II.C | - | 1 | + | - | - | - | - | - | - | 5.13 |
| **18** | 32 | non-seminoma | testis | - | - | II.A | - | 1 | + | - | - | - | - | - | - | 4.98 |
| **19** | 24 | non-seminoma | testis | - | - | II.A | - | 1 | + | - | - | - | - | - | - | 4.17 |
| **20** | 47 | non-seminoma | testis | - | - | II.A | - | 1 | + | - | - | - | - | - | - | 3.43 |
| **21** | 53 | seminoma | testis | - | - | II.B | - | 1 | + | - | - | - | - | - | - | 5.69 |
| **22** | 18 | non-seminoma | testis | - | - | II.B | - | 1 | + | - | - | - | - | - | - | 3.06 |
| **23** | 20 | non-seminoma | testis | - | - | II.B | - | 2 | + | - | - | - | - | - | - | 3.06 |
| **24** | 40 | seminoma | testis | - | - | II.B | - | 1 | + | - | - | - | - | - | - | 4.81 |
| **25** | 38 | seminoma | testis | - | - | II.B | - | 1 | + | - | - | - | - | - | - | 6.89 |
| **26** | 27 | seminoma | testis | - | - | II.C | - | 1 | + | - | - | - | - | - | - | 3.36 |
| **27** | 57 | seminoma | testis | - | - | III.A | - | 1 | + | - | - | - | - | - | - | 5.89 |
| **28** | 31 | non-seminoma | retroperitoneum | - | - | III.B | - | 2 | + | - | - | - | - | - | - | 2.98 |
| **29** | 36 | seminoma | testis | - | - | III.A | - | 1 | + | - | - | - | - | - | - | 3.34 |
| **30** | 43 | non-seminoma | testis | - | - | III.A | - | 1 | - | - | + | - | - | - | - | 5.22 |
| **31** | 47 | seminoma | testis | - | - | III.A | - | 1 | + | - | + | - | - | - | - | 3.43 |
| **32** | 33 | non-seminoma | testis | - | - | III.A | - | 1 | + | - | + | - | - | - | - | 3.48 |
| **33** | 35 | non-seminoma | testis | - | - | III.A | - | 1 | + | + | + | - | - | - | - | 5.26 |
| **34** | 25 | non-seminoma | testis | - | - | III.A | - | 1 | + | - | + | - | - | - | - | 2.17 |
| **35** | 25 | non-seminoma | testis | - | - | III.B | - | 2 | + | - | - | - | - | + | - | 3.04 |
| **36** | 19 | non-seminoma | testis | - | - | III.C | - | 3 | + | + | - | - | - | - | + | 5.01 |
| **37** | 20 | non-seminoma | testis | - | - | I.S | - | 1 | - | - | - | - | - | - | - | 4.16 |
| **38** | 24 | non-seminoma | testis | - | - | I.S | - | 1 | - | - | - | - | - | - | - | 3.10 |
| **39** | 43 | seminoma | testis | + | - | III.B | - | 2 | + | + | + | - | - | - | - | 7.16 |
| **40** | 34 | non-seminoma | testis | - | - | I.A | - | 1 | - | - | - | - | - | - | - | 4.17 |
| **41** | 41 | non-seminoma | testis | - | - | I.B | - | 1 | - | - | - | - | - | - | - | 3.23. |
| **42** | 32 | non-seminoma | testis | - | - | II.B | - | 1 | + | - | - | - | - | - | - | 6.50 |
| **43** | 22 | non-seminoma | testis | - | - | II.B | - | 1 | + | - | - | - | - | - | - | 37.8 |
| **44** | 39 | non-seminoma | testis | - | - | II.B | - | 1 | + | - | - | - | - | - | - | 3.39 |
| **45** | 34 | Seminoma | testis | - | - | II.C | + | 1 | + | - | - | - | - | - | - | 4.43 |
| **46** | 60 | seminoma | retroperitoneum | - | - | II.C | - | 1 | + | - | - | - | - | - | - | 3.74 |
| **47** | 31 | non-seminoma | testis | - | - | II.A | - | 1 | + | - | - | - | - | - | - | 7.01 |
| **48** | 21 | Seminoma | testis | - | - | II.C | - | 1 | + | - | - | - | - | - | - | 3.42 |
| **49** | 38 | Seminoma | testis | - | - | III.A | - | 1 | - | + | - | - | - | - | - | 3.81 |
| **50** | 59 | seminoma | testis | - | - | III.B | - | 1 | + | - | - | - | - | - | - | 4.69 |
| **51** | 33 | non-seminoma | testis | - | - | III.B | - | 2 | + | - | - | - | - | - | - | 4.30 |
| **52** | 56 | non-seminoma | retroperitoneum | - | - | III.B | - | 2 | + | - | - | - | - | - | - | 4.79 |
| **53** | 20 | non-seminoma | testis | - | - | III.B | - | 2 | + | - | - | - | - | - | - | 3.49 |
| **54** | 27 | non-seminoma | testis | - | - | II.A | - | 1 | + | - | - | - | - | - | - | 7.45 |
| **55** | 43 | seminoma | testis | - | - | II.C | + | 1 | + | - | - | - | - | - | - | 6.99 |
| **56** | 25 | non-seminoma | mediastinum | + | + | III.C | - | 3 | - | + | - | - | - | - | - | 9.46 |
| **57** | 32 | seminoma | testis | - | - | III.A | + | 1 | - | - | - | - | - | - | - | 4.02 |
| **58** | 49 | non-seminoma | testis | - | - | I.B | - | 1 | - | - | - | - | - | - | - | 3.71 |
| **59** | 58 | non-seminoma | testis | - | - | I.B | - | 1 | - | - | - | - | - | - | - | 5.02 |
